# Supplementary material for: Unveiling the Bioactive Potential of the Invasive Jellyfish Phyllorhiza punctata Through Integrative Transcriptomic and Proteomic Analyses
Source: Biomolecules. 2025 Aug 4;15(8):1121. doi: 10.3390/biom15081121 (PMC12383608; doi:10.3390/biom15081121)
Supplement: Supplementary file 1 [file biomolecules-15-01121-s001.zip › Suplementary Material/FileS3/PEPFOLD4/XP_065052055_12663/clusters.html]

 PEP-FOLD clusters


Download archive of all models  here   

|  |  |  |
| --- | --- | --- |
| # | file | sOPEP |
| # cluster 1 - Size: 15 |  |  |
|  | XP\_065052055\_12663-model1 | -15.713 |
|  | XP\_065052055\_12663-model1.1 | -15.713 |
|  | XP\_065052055\_12663-model1.2 | -15.713 |
|  | XP\_065052055\_12663-model1.3 | -15.6592 |
|  | XP\_065052055\_12663-model1.4 | -15.6592 |
|  | XP\_065052055\_12663-model1.5 | -15.6592 |
|  | XP\_065052055\_12663-model1.6 | -15.6592 |
|  | XP\_065052055\_12663-model1.7 | -15.6592 |
|  | XP\_065052055\_12663-model1.8 | -15.6592 |
|  | XP\_065052055\_12663-model1.9 | -15.5644 |
|  | XP\_065052055\_12663-model1.10 | -15.5644 |
|  | XP\_065052055\_12663-model1.11 | -15.5644 |
|  | XP\_065052055\_12663-model1.12 | -15.5644 |
|  | XP\_065052055\_12663-model1.13 | -15.5644 |
|  | XP\_065052055\_12663-model1.14 | -15.5644 |
| # cluster 2 - Size: 1 |  |  |
|  | XP\_065052055\_12663-model2 | -15.6778 |
| # cluster 3 - Size: 6 |  |  |
|  | XP\_065052055\_12663-model3 | -15.6266 |
|  | XP\_065052055\_12663-model3.1 | -15.6266 |
|  | XP\_065052055\_12663-model3.2 | -15.6266 |
|  | XP\_065052055\_12663-model3.3 | -15.6266 |
|  | XP\_065052055\_12663-model3.4 | -15.6266 |
|  | XP\_065052055\_12663-model3.5 | -15.3628 |
| # cluster 4 - Size: 9 |  |  |
|  | XP\_065052055\_12663-model4 | -15.6188 |
|  | XP\_065052055\_12663-model4.1 | -15.6188 |
|  | XP\_065052055\_12663-model4.2 | -15.6188 |
|  | XP\_065052055\_12663-model4.3 | -15.5505 |
|  | XP\_065052055\_12663-model4.4 | -15.5192 |
|  | XP\_065052055\_12663-model4.5 | -15.5192 |
|  | XP\_065052055\_12663-model4.6 | -15.5192 |
|  | XP\_065052055\_12663-model4.7 | -15.4735 |
|  | XP\_065052055\_12663-model4.8 | -15.3213 |
| # cluster 5 - Size: 8 |  |  |
|  | XP\_065052055\_12663-model5 | -15.6018 |
|  | XP\_065052055\_12663-model5.1 | -15.6018 |
|  | XP\_065052055\_12663-model5.2 | -15.6018 |
|  | XP\_065052055\_12663-model5.3 | -15.4395 |
|  | XP\_065052055\_12663-model5.4 | -15.4395 |
|  | XP\_065052055\_12663-model5.5 | -15.4395 |
|  | XP\_065052055\_12663-model5.6 | -15.4395 |
|  | XP\_065052055\_12663-model5.7 | -15.4395 |
| # cluster 6 - Size: 4 |  |  |
|  | XP\_065052055\_12663-model6 | -15.5924 |
|  | XP\_065052055\_12663-model6.1 | -15.5396 |
|  | XP\_065052055\_12663-model6.2 | -15.4462 |
|  | XP\_065052055\_12663-model6.3 | -15.4462 |
| # cluster 7 - Size: 34 |  |  |
|  | XP\_065052055\_12663-model7 | -15.5716 |
|  | XP\_065052055\_12663-model7.1 | -15.5716 |
|  | XP\_065052055\_12663-model7.2 | -15.5716 |
|  | XP\_065052055\_12663-model7.3 | -15.5284 |
|  | XP\_065052055\_12663-model7.4 | -15.5284 |
|  | XP\_065052055\_12663-model7.5 | -15.5284 |
|  | XP\_065052055\_12663-model7.6 | -15.5284 |
|  | XP\_065052055\_12663-model7.7 | -15.5284 |
|  | XP\_065052055\_12663-model7.8 | -15.5284 |
|  | XP\_065052055\_12663-model7.9 | -15.5284 |
|  | XP\_065052055\_12663-model7.10 | -15.5284 |
|  | XP\_065052055\_12663-model7.11 | -15.5284 |
|  | XP\_065052055\_12663-model7.12 | -15.5284 |
|  | XP\_065052055\_12663-model7.13 | -15.5284 |
|  | XP\_065052055\_12663-model7.14 | -15.5284 |
|  | XP\_065052055\_12663-model7.15 | -15.5284 |
|  | XP\_065052055\_12663-model7.16 | -15.5284 |
|  | XP\_065052055\_12663-model7.17 | -15.5284 |
|  | XP\_065052055\_12663-model7.18 | -15.5284 |
|  | XP\_065052055\_12663-model7.19 | -15.5284 |
|  | XP\_065052055\_12663-model7.20 | -15.5284 |
|  | XP\_065052055\_12663-model7.21 | -15.5284 |
|  | XP\_065052055\_12663-model7.22 | -15.5284 |
|  | XP\_065052055\_12663-model7.23 | -15.4517 |
|  | XP\_065052055\_12663-model7.24 | -15.4444 |
|  | XP\_065052055\_12663-model7.25 | -15.4444 |
|  | XP\_065052055\_12663-model7.26 | -15.4444 |
|  | XP\_065052055\_12663-model7.27 | -15.4444 |
|  | XP\_065052055\_12663-model7.28 | -15.4444 |
|  | XP\_065052055\_12663-model7.29 | -15.4444 |
|  | XP\_065052055\_12663-model7.30 | -15.4444 |
|  | XP\_065052055\_12663-model7.31 | -15.4112 |
|  | XP\_065052055\_12663-model7.32 | -15.4112 |
|  | XP\_065052055\_12663-model7.33 | -15.3776 |
| # cluster 8 - Size: 16 |  |  |
|  | XP\_065052055\_12663-model8 | -15.5272 |
|  | XP\_065052055\_12663-model8.1 | -15.5272 |
|  | XP\_065052055\_12663-model8.2 | -15.5272 |
|  | XP\_065052055\_12663-model8.3 | -15.5272 |
|  | XP\_065052055\_12663-model8.4 | -15.5272 |
|  | XP\_065052055\_12663-model8.5 | -15.5272 |
|  | XP\_065052055\_12663-model8.6 | -15.5272 |
|  | XP\_065052055\_12663-model8.7 | -15.5272 |
|  | XP\_065052055\_12663-model8.8 | -15.5272 |
|  | XP\_065052055\_12663-model8.9 | -15.5272 |
|  | XP\_065052055\_12663-model8.10 | -15.5272 |
|  | XP\_065052055\_12663-model8.11 | -15.5272 |
|  | XP\_065052055\_12663-model8.12 | -15.5272 |
|  | XP\_065052055\_12663-model8.13 | -15.4397 |
|  | XP\_065052055\_12663-model8.14 | -15.4397 |
|  | XP\_065052055\_12663-model8.15 | -15.4397 |
| # cluster 9 - Size: 2 |  |  |
|  | XP\_065052055\_12663-model9 | -15.5029 |
|  | XP\_065052055\_12663-model9.1 | -15.4037 |
| # cluster 10 - Size: 3 |  |  |
|  | XP\_065052055\_12663-model10 | -15.4689 |
|  | XP\_065052055\_12663-model10.1 | -15.4689 |
|  | XP\_065052055\_12663-model10.2 | -15.3341 |
| # cluster 11 - Size: 1 |  |  |
|  | XP\_065052055\_12663-model11 | -15.414 |
| # cluster 12 - Size: 1 |  |  |
|  | XP\_065052055\_12663-model12 | -15.3997 |
|  |  |  |
